# Phlegmasia Cerulea Dolens: A New Perspective on Management

**DOI:** 10.7759/cureus.16257

**Published:** 2021-07-08

**Authors:** Harry G Sequeira Gross, Yomary Jimenez, Camelia Ciobanu, Kidist Tarekegn, Ana Colon Ramos, Jeffrey Lazar

**Affiliations:** 1 Internal Medicine, St. Barnabas Hospital, The Bronx, USA; 2 Medicine, St. Barnabas Hospital, The Bronx, USA; 3 Emergency Medicine, St. Barnabas Hospital Health System, The Bronx, USA

**Keywords:** phlegmasia cerulea dolens, unexpected outcome, case presentation, new perspective, rare case

## Abstract

This is a case of phlegmasia cerulea dolens (PCD) with unexpected but complete resolution of symptoms with short-term administration of heparin products, despite falling into category IIb according to the Rutherford limb ischemia scale, which regularly requires aggressive surgical intervention.

We present a case of a 58-year-old Hispanic female with a past medical history of gastritis who arrived at the emergency room (ER) with acute onset severe pain on the left leg associated with discoloration of the leg. The patient was quickly diagnosed with PCD affecting the left lower extremity, which quickly resolved after administration of heparin infusion for one hour, despite the degree of limb ischemia.

There is no consensus for the treatment of this condition. The most interesting feature of this case is the prompt resolution of symptoms with short-term administration of anticoagulation with total resolution without the need for thrombolysis or thrombectomy. This may suggest that prompt pharmacologic treatment in patients eligible for anticoagulation may successfully restore venous flow negating the need for further intervention.

## Introduction

Phlegmasia cerulea dolens (PCD) is a massive deep vein thrombosis (DVT) that affects the major and collateral extremity veins [1]. This extensive DVT leads to severe, purple discoloration and edema of the limb. Hence the name, which translates to painful blue edema. According to some authors [2], this entity can lead to irreversible venous gangrene in 40%-60% of cases, and without timely management and/or if left untreated can lead to 25%-40% mortality [3-5].

There are several risk factors that have been associated with the development of PCD. The most common ones are malignancy, hypercoagulable state, history of DVT, prolonged immobility, and a variety of inflammatory conditions. However, 10%-16% of the patients have no known risk factors [2].

According to Lee and Hsieh [2], therapeutic strategies depend on the clinical picture and the extent of tissue ischemia and venous gangrene.

## Case presentation

**Figure 1:**
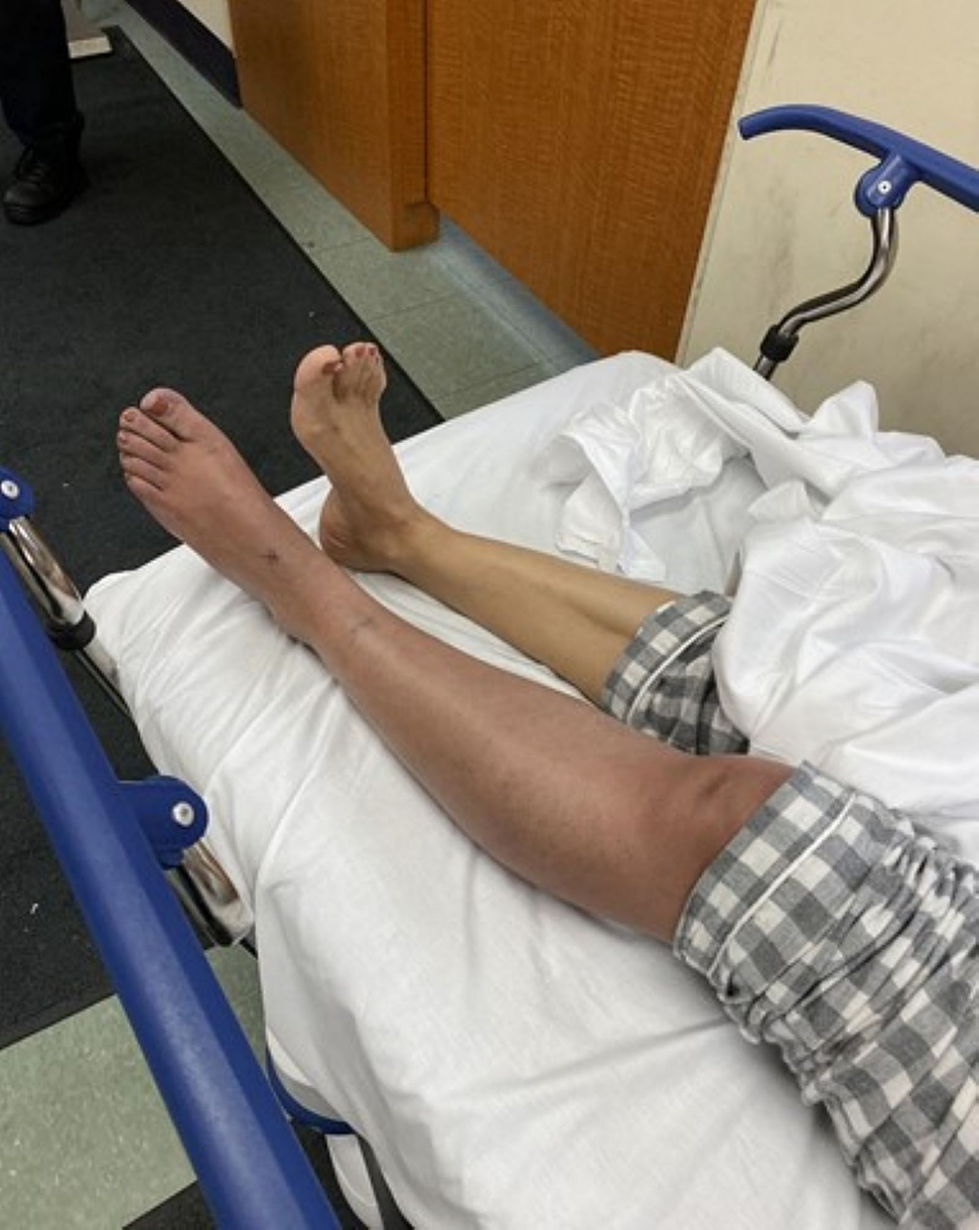
The patient's limb on presentation to the ER. ER, emergency room

A Hispanic 58-year-old female with a past medical history of gastritis arrived into the emergency room (ER) with acute onset severe pain on the left leg associated with discoloration of the leg. According to the patient, approximately three hours prior to her arrival to the ER, she was walking when, suddenly, she started experiencing sharp pain in her left leg which rapidly progressed. The physical examination was remarkable for left lower extremity pain, discoloration, edema, associated with tenderness to touch, diminished distal pulses, and reduce sensation and mobility throughout the leg, which can be appreciated below (Figure 1).

**Figure 2:**
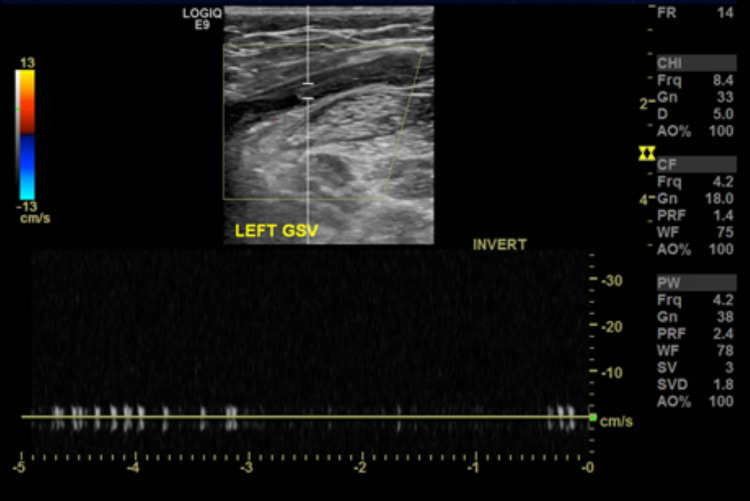
Doppler US from the affected limb. US, ultrasound

**Figure 3:**
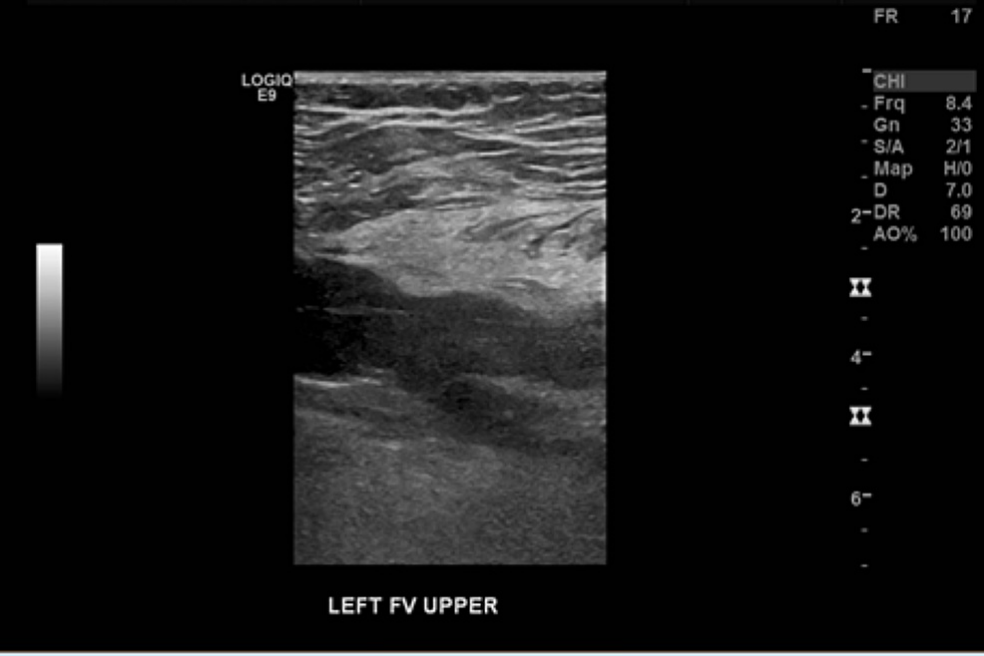
Aorto-ilio-femoral runoff with delayed phase was performed on the affected limb.

Within the first 30 min after her arrival to the hospital, a pressure Doppler was performed where extensive deep vein thrombosis (DVT) (left femoral-popliteal venous thrombosis) (Figure 2) diagnosis was made and within the subsequent 60 min, a computed tomography angiography (CTA) aorto-ilio-femoral runoff with delayed phase was performed, showing delayed opacification of arterial segments below the knee level (Figure 3), confirming the diagnosis. A bolus of heparin was given, followed by an infusion. Thrombolysis was offered to the patient, however, she refused due to concern for side effects.

 

After one hour of starting heparin infusion, blood flow to the leg was regained and the patient recovered full sensation and movement in the leg; coloration returned to normal in the affected leg. The patient was admitted for continued observation and discharged later on direct oral anticoagulants (DOACs) without any recurrence of DVT so far.

The patient denied recent flights, a sedentary lifestyle, surgery, trauma, alcohol, tobacco, and drug use. There was neither a previous episode nor a family history of similar symptoms to her knowledge. Age-appropriate oncology screening was negative; mammogram from June 2020 was normal; colonoscopy four years before the presentation was also negative; Pap smears as well. Thrombophilia workup done by the Hematology/Oncology service was unrevealing, leading to a diagnosis of unprovoked DVT.

The curious part of the case is that even though thrombolysis and thrombectomy are considered the gold standard in the treatment of PCD, this patient had a spontaneous recovery only with heparin which serves as the only purpose to prevent the propagation of the thrombus. This specific patient according to Rutherford limb ischemia classification [6] was a IIb which granted surgical intervention. Still, it remains unclear how such an extensive DVT improved in so quick fashion without thrombectomy/thrombolysis but seems that early use of heparin might have played an important role in the evolution of this patient's case and that early use of heparin might decrease the need for surgical intervention in some patients.

## Discussion

Phlegmasia cerulea dolens is a rare entity that refers to a painful, edematous, and cyanotic limb due to a massive DVT. The pain is due to venous hypertension with increased compartment pressure within the extremity. The cyanotic appearance of the limb is secondary to carboxyhemoglobin build-up in dermal venous plexuses, as a result of a multilevel occlusion [1].

This entity represents a major therapeutic challenge because there is no consensus regarding the treatment. According to Lee and Hsieh [2], therapeutic strategies depend on the clinical picture and the extent of tissue ischemia and venous gangrene. They favor that, in any case, the goal of management should be to prevent thrombus propagation and to preserve collateral circulation and tissue viability.

Weaver et al. [7] describe three therapeutic measures for the management of PCD: IV heparin, venous thrombectomy, and thrombolytic therapy, and more recently oral anticoagulants like argatroban have been described to be effective under special circumstances by other authors [8]. Their data suggest that non-gangrenous forms of PCD have a successful response to systemic anticoagulation.

This was evident in our patient, who received IV heparin infusion with rapid improvement of the symptoms. In addition, they describe that combination therapy with venous thrombectomy and heparin is beneficial for severe ischemia or failure of PCD to improve after 6-12 h of heparin. On the other hand, there are other authors who favor a surgical approach. Lee [2] describes fasciotomy for those who develop compartment syndrome, while some new data suggest there might be a place for oral anticoagulation in the right patient [9], but only until surgical intervention is no longer a viable option for the patient. 

## Conclusions

Phlegmasia cerulea dolens remains a rare entity and, therefore, building a strong evidence-based approach has been very complicated to obtain. Adequately stratifying patients to decide the treatment has proven to be as important as administering the treatment in a timely manner. In our case, complete resolution of the symptoms was achieved with short-term administration of anticoagulation without the need for thrombolysis or thrombectomy despite the high degree of limb ischemia.

There is no consensus for the treatment of this condition and, with time, management of PCD has not changed significantly but, the sequence of events observed in this patient suggests that not only the degree of limb ischemia should be considered to decide management, but also the duration of symptoms. In our patient, the use of heparin products proved to be beneficial. In addition, some evidence suggests that the use of novel oral anticoagulants (NOACs) may play an important role in a subgroup of patients. More cases are needed to build and solidify current information about all treatment options.
